# Army Notes

**Published:** 1899-05

**Authors:** 


### ARMY NOTES.

Candidates of the veterinary profession for appointment under the new rules regulating the selection of army veterinarians will be subjected to a basis examination and a subject examination. The first will embrace English grammar, arithmetic, geography (North America), history (United States), and will have a relative value of 20 per cent. of the total examination.

The subject examination will embrace anatomy, pathology, practice of medicine, descriptive and operative surgery, materia medica and therapeutics, sanitary medicine, conformation and examination for soundness, horseshoeing, meat-inspection, veterinary hygiene (including general feeding and watering), stabling (garrison and field), saddling (pack and draught), bitting, aptitude, and general efficiency.

Only the veterinarians now employed in the army will be admitted to the first examination, and if any vacancy remains a similar examination will be prepared for civilian veterinarians who wish to enter the army.

In all probability the officers will endeavor to favor those who pass the examination and are appointed to the position provided by the last Congress.

Captains Steever and Johnson, Third Cavalry, United States Army, and Dr. Victor Norgaard, V.S., Bureau of Animal Industry, United States Department of Agriculture, have been detailed at Washington, D. C., to formulate questions and conduct the examinations for veterinarians in the United States Army.

